# Branched-Chain Amino Acids and Di-Alanine Supplementation in Aged Mice: A Translational Study on Sarcopenia

**DOI:** 10.3390/nu15020330

**Published:** 2023-01-09

**Authors:** Paola Mantuano, Brigida Boccanegra, Gianluca Bianchini, Ornella Cappellari, Lisamaura Tulimiero, Elena Conte, Santa Cirmi, Francesca Sanarica, Michela De Bellis, Antonietta Mele, Antonella Liantonio, Marcello Allegretti, Andrea Aramini, Annamaria De Luca

**Affiliations:** 1 Section of Pharmacology, Department of Pharmacy-Drug Sciences, University of Bari “Aldo Moro”, Orabona 4—Campus, 70125 Bari, Italy; 2 Research & Early Development, Dompé farmaceutici S.p.A., Via Campo di Pile, s.n.c. 67100 L’Aquila, Italy

**Keywords:** sarcopenia, aging, aged C57BL/6J mice, branched-chain amino acids, L-Alanine, L-Alanyl-L-Alanine

## Abstract

In age-related sarcopenia, the gradual loss of skeletal muscle mass, function and strength is underpinned by an imbalanced rate of protein synthesis/breakdown. Hence, an adequate protein intake is considered a valuable strategy to mitigate sarcopenia. Here, we investigated the effects of a 12-week oral supplementation with branched-chain amino acids (BCAAs: leucine, isoleucine, and valine) with recognized anabolic properties, in 17-month-old (AGED) C57BL/6J male mice. BCAAs (2:1:1) were formulated in drinking water, alone or plus two L-Alanine equivalents (2ALA) or dipeptide L-Alanyl-L-Alanine (Di-ALA) to boost BCAAs bioavailability. Outcomes were evaluated on in/ex vivo readouts vs. 6-month-old (ADULT) mice. In vivo hind limb plantar flexor torque was improved in AGED mice treated with BCAAs + Di-ALA or 2ALA (recovery score, R.S., towards ADULT: ≥20%), and all mixtures significantly increased hind limb volume. Ex vivo, myofiber cross-sectional areas were higher in gastrocnemius (GC) and soleus (SOL) muscles from treated mice (R.S. ≥ 69%). Contractile indices of isolated muscles were improved by the mixtures, especially in SOL muscle (R.S. ≥ 20%). The latter displayed higher mTOR protein levels in mice supplemented with 2ALA/Di-ALA-enriched mixtures (R.S. ≥ 65%). Overall, these findings support the usefulness of BCAAs-based supplements in sarcopenia, particularly as innovative formulations potentiating BCAAs bioavailability and effects.

## 1. Introduction

Sarcopenia, defined as a progressive decline in skeletal muscle mass, strength, and function, is recognized as a critical clinical condition underlying frailty and disability in elderly people, with serious consequences for their quality of life and longevity [1,2].

Although the aetiology of sarcopenia is not yet fully understood, multiple age-related causes and risk factors (i.e., reduced physical activity and nutritional intake) [1] have been identified in patients, as well as in aged rodents (rats and mice). These latter are considered robust and reliable animal models because they exhibit the natural aging process, with symptoms and outcomes similar to those found in humans [3]. Skeletal muscles from patients aged 65 and over [2,3,4] and old rodents ranging from 20 to 24 months of age [3,5,6] share a common atrophic phenotype, featuring a substantial reduction in skeletal muscle mass and myofiber cross-sectional area (CSA), resulting from a disruption in protein homeostasis tilting the equilibrium between protein synthesis and breakdown. In sarcopenic models, this has been primarily linked to an overactivation of the Ubiquitin (Ub)–proteasome system, with the FoxO3-mediated upregulation of atrophy-related players, such as muscle-specific E3 Ub-protein ligases muscle atrophy F-box (MAFbx)/Atrogin-1 and muscle RING-finger 1 (MuRF1) [6]. These are, in turn, responsible for the degradation of several proteins, including structural myosin heavy chains (MyHCs) and myogenic regulatory factors (e.g., MyoD and myogenin) [7]. Similarly, inefficiencies in the IGF1-Akt/mammalian target of the rapamycin (mTOR) axis, a major regulatory pathway for protein synthesis, have been reported in old humans and rodents [2,6,8,9]. In addition, aged mice muscles show alterations in mitochondrial function and biogenesis through the deactivation of the AMP kinase (AMPK)–Sirtuin1 (SIRT1)–Peroxisome proliferator-activated receptor-γ coactivator (PGC)-1α energy-sensing pathway, which may account for reactive oxygen species (ROS)-related oxidative stress and inflammatory cytokine release, possibly worsening the sarcopenic phenotype [6,10].

Despite the remarkable progress made in the understanding of sarcopenia pathogenesis, no effective pharmacological remedies exist yet to halt or prevent age-related muscle-wasting [11,12]. Given the increasing population of older adults worldwide, this clearly represents a high unmet medical need. To date, the combination of proper nutrition and physical activity is considered the most effective strategy to prevent and/or mitigate age-related sarcopenia [11,12].

In particular, the amino acid composition of dietary proteins has a great impact on skeletal muscle protein metabolism, and the ingestion of an adequate protein amount preserves muscle functionality, especially in advanced age [12,13]. Importantly, older muscles can still mount a response to exogenously administered amino acids, particularly essential amino acids (EAAs) [5,14], supporting the interest for the dietary supplementation of amino acids with anabolic properties in sarcopenia.

Among EAAs, branched-chain amino acids (BCAAs: leucine, isoleucine, and valine) are claimed to have the highest anabolic potential, because BCAAs (especially leucine) directly stimulate protein synthesis by activating the mTOR pathway [15]. Similarly, BCAAs inhibit muscle protein catabolism by reducing the expression of Atrogin-1 and MuRF-1 and promote insulin secretion and glucose uptake in skeletal muscle, which is crucial to supply energetic substrates for anabolic reactions in models of atrophy [16]. Moreover, BCAAs supplementation appears to improve lifespan, mitochondrial biogenesis (via SIRT1) and protein synthesis (via mTOR) in middle-aged mice, and to provide benefits for muscle mass, strength and protein synthesis rates in older subjects with pre-sarcopenia or sarcopenia [17,18,19]. In this frame, we recently demonstrated that a 4-week oral supplementation with BCAAs (in a 2:1:1 ratio) can preserve myofiber CSA, total protein content and mass of postural soleus (SOL) muscle in a murine model of hind limb unloading (HU)-induced disuse atrophy [20]. These results were noteworthy, especially when BCAAs were combined with two L-Alanine equivalents (2ALA) or with the dipeptide L-Alanyl-L-Alanine (Di-ALA). L-ALA is indeed the main amino acid derived from BCAAs catabolism, and we disclosed that, with a dose of 2ALA, it boosts BCAAs bioavailability and its ergogenic effect in trained adult animals [21]. In addition, the formulation combining BCAAs with Di-ALA tested in HU mice is advantageous, because the dipeptide can increase L-ALA bioavailability and residence time, due to faster rates of intestinal uptake and absorption with respect to free amino acids [20].

In light of these encouraging findings, this study sought to gain novel insights into the potential benefits of innovative formulations that combine BCAAs with boosting molecules, 2ALA or Di-ALA, in the context of age-related sarcopenia.

The potential efficacy and safety of BCAAs, alone or plus 2ALA/Di-ALA, were evaluated via a clinically oriented experimental approach combining multiple in vivo and ex vivo disease-relevant readouts to be assessed in C57BL/6J aged mice.

## 2. Materials and Methods

This study was approved by the National Ethics Committee for Research Animal Welfare of the Italian Ministry of Health (authorization no. 1119/2020-PR). The experimental protocol was designed and carried out in compliance with the Italian Guidelines for Care and Use of Laboratory Animals (D.L.116/92) and the European Directive (2010/63/EU), as well as with the ARRIVE Guidelines, the 1964 Declaration of Helsinki and its later amendments. As sarcopenia is a muscular disease, the rigor of in vivo and ex vivo experiments was inspired by the international guidelines for preclinical studies in neuromuscular disorders (NMDs) (http://www.treat-nmd.eu/research/preclinical/dmd-sops/ accessed on 01 December 2022) [22].

### 2.1. Animals and Treatments

A total of 32, 17-month-old male C57BL/6J WT mice (AGED) and 6, 3-month-old male C57BL/6J WT mice (ADULT) were purchased from The Jackson Laboratory (USA, distributed by Charles River Laboratories, Calco, Italy).

All mice were acclimatized for ~1 week in our animal facility before the beginning of the experimental protocol, housed in suitable cages (3–4 mice per cage) with appropriate temperature (22–24 °C), humidity (50–60%) and light/dark cycle (12 h/12 h) conditions [20,21]. After acclimatization, AGED mice cohorts (4 groups of n = 8, each), which were homogeneous for body mass (BM) and forelimb force, were randomly assigned to each treatment condition, namely the vehicle (filtered tap water), BCAAs or BCAAs combined with 2ALA or Di-ALA. The n = 6 ADULT mice were used as the control group to assess aging-associated sarcopenia outcomes. A sample size was chosen as the best compromise to guarantee a robust statistical relevance, considering both old mice frailty and a long treatment duration (12 weeks, T0–T12).

Every week, each formulation was freshly prepared by dissolving the amino acid mixture powder in filtered tap water to obtain the intended final dose, according to mice average BM and water consumption [23]. Table 1 shows compositions (in weight ratios) and final doses (in mg/kg). These latter translate to equivalent doses in humans, according to an appropriate calculation method for dose conversion [24]. All mice were fed a daily amount of 5 g/mouse chow (VRF1 standard pelleted diet, Charles River Laboratories) [20,21,23].

### 2.2. In Vivo Monitoring and Functional Tests

All animals were regularly checked for health and well-being throughout the study using adequate care in mice handling to avoid discomfort or stress during in vivo measurements [20,21,22,23,25]. None of the cohorts exhibited signs of pain, distress, or macroscopic alterations in vital functions. Mice body masses were measured on a weekly basis.

#### 2.2.1. Forelimb Grip Strength and Isometric Plantar Flexor Torque

Forelimb force was assessed every two weeks, from T0 to T12, via a grip strength meter (Columbus Instruments, Columbus, OH, USA), according to a standard procedure [20,21,23,26,27]. The maximal force, both absolute (kg force, KGF) and normalized to BM (KGF/kg), obtained from five measurements per mouse, was used for data analysis.

At T0 and T12, the isometric torque produced by hind limb plantar flexors (gastrocnemius—GC, soleus—SOL, and plantaris muscles) was assessed via the 1300A 3-in-1 Whole Animal System (Aurora Scientific Inc.—ASI, Aurora, ON, Canada) in mice under isoflurane inhalation anaesthesia, adequately prepared and positioned on a temperature-controlled platform (mod. 809B, ASI) at 36 °C with a footplate connected to a dual-mode servomotor (mod. 300C-LR, ASI) [20,21,26,27]. Contractions were elicited via percutaneous electrical stimulation of the sciatic nerve, using a pair of needle electrodes (Chalgren Enterprises Inc., CA, USA) connected to a high-power bi-phase stimulator (mod. 701C, ASI), in turn controlled by a data acquisition signal interface (mod. 604A, ASI) and by ASI Dynamic Muscle Control software (DMCv5.415). After adjusting the current, a series of isometric contractions was recorded at increasing frequencies (pulses of 200 ms, from 1 to 200 Hz, one every 30 s). Data for plantar flexor torque (N·cm) recorded at each frequency were obtained via ASI Dynamic Muscle Analysis software (DMAv5.201), normalized to each mouse’s BM (N·mm/kg) and used to generate torque–frequency curves [20,21,26,27].

#### 2.2.2. Ultrasonography

Hind limb volume was non-invasively measured at T0 and T12 via the ultra-high frequency ultrasound biomicroscopy system Vevo^®^ 2100 (VisualSonics, Toronto, ON, Canada). Each mouse, put under inhalation anaesthesia with isoflurane, was placed on a thermostatically controlled platform (37 °C) in a ventral decubitus position and was prepared for the imaging session [21]. A three-dimensional (3D) volume scan was acquired by translating the probe parallel to the long axis of each hind limb. Two-dimensional (2D) images were acquired at regular intervals via a MS250 probe at 21 MHz, with lateral and axial resolutions, respectively, of 165 and 75 µm. Three-dimensional images were reconstructed from multiple 2D frames, visualized with VisualSonics 3D software, and used to calculate hind limb total volume (in mm^3^), which was then normalized to BM. For each mouse, three 2D images showing GC and SOL muscles were selected to obtain echodensity as an index of possible fat and fibrous tissue infiltration by adapting the protocol from [28]. Echodensity was measured using ImageJ^®^ software by creating a grey scale analysis histogram on a defined constant muscle section in pixels. For each mouse, muscle echodensity was calculated as the main value obtained from 4 frames of the same acquisition, drawing the regions of interest in the same area of each muscle among mice. During each session, measurements of diaphragm (DIA) movement amplitude (mm) and echodensity were also performed [28].

### 2.3. Ex Vivo Procedures

#### 2.3.1. Sample Harvesting, Processing and Storage

In vivo monitoring was followed by ex vivo experiments. In this phase, several biological samples and tissues were harvested from each mouse and were differently prepared for ex vivo muscle physiology, biochemistry, molecular biology, and histology. The time-consuming nature of ex vivo physiology measurements allowed us to sacrifice a maximum of 2–3 animals per day, requiring an extra time window of ~3 weeks. Mice were treated until the day of sacrifice; hence, T12 was considered the final time point for in vivo data analysis. Animals from each cohort were equally distributed over time during ex vivo procedures to avoid any bias due to different exposure to treatments [27].

Mice were anesthetized via intraperitoneal (IP) injection with a ketamine (100 mg/kg) and xylazine (16 mg/kg) cocktail. If necessary, a boost of 30 mg/kg ketamine was injected to ensure a longer, deeper sedation. After the onset of anaesthesia, pilocarpine hydrochloride (1 mg/kg, Sigma-Aldrich, St. Louis, MO, USA) was injected via IP to induce salivation; after ~5 min, saliva was collected from the oral cavity and was processed [20,21] to measure salivary immunoglobulin A (IgA) levels via an enzyme-linked immunosorbent assay (ELISA).

Extensor digitorum longus (EDL) and soleus (SOL) muscles from one hind limb and a portion of the right hemi-diaphragm (DIA) were carefully isolated and prepared to be used fresh for contractile recordings, as described later. After this, EDL, and SOL muscles, as well as gastrocnemius (GC) muscle from one hind limb and a portion of DIA, were weighed, embedded in a small amount of Tissue-Tek^®^ optimum cutting temperature, O.C.T. (Bio-Optica, Milan, Italy), immersed in isopentane cooled with liquid nitrogen (N_2_) for 60 s and stored at −80 °C until being processed for histology. Contralateral SOL muscle was isolated, weighed, snap frozen in N_2_ and stored at −80 °C until use for the mTOR ELISA test. Spare EDL and GC muscles, as well as tibialis anterior (TA), quadriceps (QUAD) and triceps (TRI) muscles from both limbs, white and brown adipose tissue (WAT and BAT) and vital organs (liver, heart, kidneys, spleen, and brain) were harvested and weighed for a gross examination of toxicity and/or effects.

Blood samples were collected via cardiac puncture and were processed to obtain platelet-poor plasma [20,21,23,26,27]. The latter was used fresh to quantify creatine kinase (CK) and lactate dehydrogenase (LDH). Specifically, CK and LDH enzymatic activity (U/L) was measured in plasma samples via commercially available diagnostic kits (CK NAC LR and LDH LR, SGM, Rome, Italy). The assays were carried out by using a spectrophotometer (Ultrospec 2100 Pro UV/Visible, Amersham Biosciences, Little Chalfont, UK) set to a wavelength of 340 nm at 37 °C, according to the manufacturer’s instructions.

#### 2.3.2. Isometric and Eccentric Contraction Recordings

A strip of DIA (~4 mm wide) was cut from the harvested muscle and then firmly tied at the rib and at the central tendon, whilst both the extensor digitorum longus (EDL) and SOL muscles were securely tied with silk suture 6–0 (Fine Science Tools Inc., Foster City, CA, USA) at proximal and distal tendons during dissection and were gently removed from the mouse. Samples were then individually allocated into a 25 mL recording chamber containing isotonic Ringer’s solution [23] at a pH of 7.2–7.4, continuously gassed with a mixture of 95% O_2_ and 5% CO_2_ and thermostatically maintained at 27 ± 1 °C. The DIA strip, secured at the bottom of a vertical muscle bath (mod. 800A, ASI), was fixed at the rib to a dual-mode muscle lever (mod. 300C-LR, ASI); EDL and SOL muscles were placed into a horizontal muscle bath (mod. 809B-25, ASI), with one tendon fixed at the bottom and the other fixed to a 300C-LR force transducer. In each bath, electrical field stimulation was obtained with two axial platinum electrodes closely flanking the muscle, connected to a high-power bi-phase stimulator (mod. 701C, ASI). Each apparatus was equipped with a data acquisition signal interface (mod. 604A, ASI) and software (DIA: DMCv4.1.6; EDL/SOL: DMCv5.415, ASI). After equilibration (~30 min), muscle preparations were stretched to their optimal length (L_0_, measured with an external calliper) [23]. Single twitch (Ptw) tension was calculated as the mean value from 5 twitches elicited by pulses of 0.2 ms every 30 s. Tetanic contractions were elicited by applying trains of 2.0 ms pulses for 350 ms (EDL), 450 ms (DIA) or 1200 ms (SOL) at increasing frequencies (from 10 to 250 Hz) every 2 min. Maximal tetanic force (P0) was generally recorded at 120–180 Hz. Then, each muscle underwent a series of 10 eccentric contractions (consisting of an initial 300 ms isometric pulse, followed by a 200 ms stretch of 10% L_0_ at a speed of 1L0 s^−1^) every 30 s. The force decay at the 5th and 10th pulses vs. the 1st pulse was calculated, as was the compliance to stretching (mN/mm^3^). Two tetanic stimuli (120 Hz, 500 ms) were elicited 5 and 15 min after the eccentric protocol to calculate force recovery. Data were analysed via ASI software DMAv5.201. Ptw and P0 values were normalized to muscle cross sectional areas according to the equation sP = P/(Mass/Lf·D), where P is the absolute tension, Mass is the muscle mass, D is the density of skeletal muscle (1.06 g/cm^3^), and Lf is obtained by multiplying L_0_ with the previously determined muscle length to fiber length ratio (SOL = 0.71, EDL = 0.44, DIA = 1).

#### 2.3.3. Muscle Histopathology and Immunofluorescence

Serial cross-sections (8 µm thick) from properly frozen muscles (SOL, GC, EDL, and a portion of DIA) were transversally cut into a cryostat microtome set at −20 °C (HM 525 NX, Thermo Fisher Scientific, Waltham, MA, USA). Classical haematoxylin and eosin staining (H&E; Bio-Optica, Milan, Italy) was used to estimate each muscle’s architecture and the possible presence of unhealthy tissue, quantified as the percentage (%) of fibrotic/necrotic/regenerated areas on the total muscle area [7,20]. Masson’s trichrome staining (Bio-Optica) was used to detect collagen (%) as an index of muscle fibrosis. Immunofluorescence (IF) staining for laminin was used on hind limb muscles (SOL, GC, and EDL) to determine the myofiber mean cross-sectional area (CSA, µm^2^) [21,26]. To rule out any artifacts due to non-perpendicular muscle inclusion or sectioning, a first qualitative random assessment of the close-to-unit value of ellipticity was performed, especially in muscle areas exhibiting less homogeneous features.

Muscle morphological features were identified using digital images, acquired with a Nikon Eclipse Ci-L microscope unit (Nikon, Tokyo, Japan) plus ImageJ software (NIH, Bethesda, MD, USA). ImageJ was also used for picture analyses of the total and constant transverse muscle, performed on 2–3 non-overlapping fields for SOL and EDL muscles at 20× magnification, 5–6 fields at 10× magnification and 10 fields at 20× magnification for GC muscle, and 4–6 fields for DIA muscle at 20× magnification.

#### 2.3.4. Enzyme-Linked Immunosorbent Assays (ELISA)

Total mTOR protein levels were determined in frozen SOL muscle tissue via the Mouse mTOR SimpleStep ELISA^®^ kit ab206311 (Abcam, Cambridge, UK) [20]. Salivary IgA levels were measured via the Mouse IgA Ready-SET-Go! ELISA kit (eBioscience, Vienna, Austria) [20,21]. Both assays were carried out according to the manufacturers’ protocols, using a Victor 3V multilabel plate reader (Perkin Elmer, Waltham, MA, USA). Values were calculated as absolute and were normalized to the total protein content (in µg; obtained via a Bradford assay) [20,21].

#### 2.3.5. Statistics

All data were expressed as the mean ± the standard error of the mean (SEM). Multiple statistical comparisons between AGED groups were performed using a one-way analysis of variance (ANOVA), with Dunnett’s test post hoc correction (°) when the null hypothesis was rejected (*p* < 0.05). Unpaired Student’s t-test was exclusively used to compare untreated AGED vs. ADULT mice (*). All data followed, with good approximation, a normal distribution, being included in the 95% confidence interval of the mean. No outliers were identified, and the exclusion of specific samples from data analyses was only due to overt technical issues during experiments [20]. The in vivo/ex vivo procedures, data collection and analysis were conducted in a blinded fashion by the experimenters.

Whenever appropriate, the recovery score (R.S.), an objective index directly indicating how much of the deficit is recovered (%) by a treatment, was calculated according to TREAT-NMD SOPs, as follows:

(1) $$\mathrm{Recovery}\;\mathrm{score}=\frac{\left(\mathrm{treated}\;\mathrm{AGED}\;\mathrm{mice}-\mathrm{untreated}\;\mathrm{AGED}\;\mathrm{mice}\right)}{\left(\mathrm{control}\;\mathrm{mice}-\mathrm{untreated}\;\mathrm{AGED}\;\mathrm{mice}\right)}$$

## 3. Results

### 3.1. In Vivo Data

**Figure 1:**
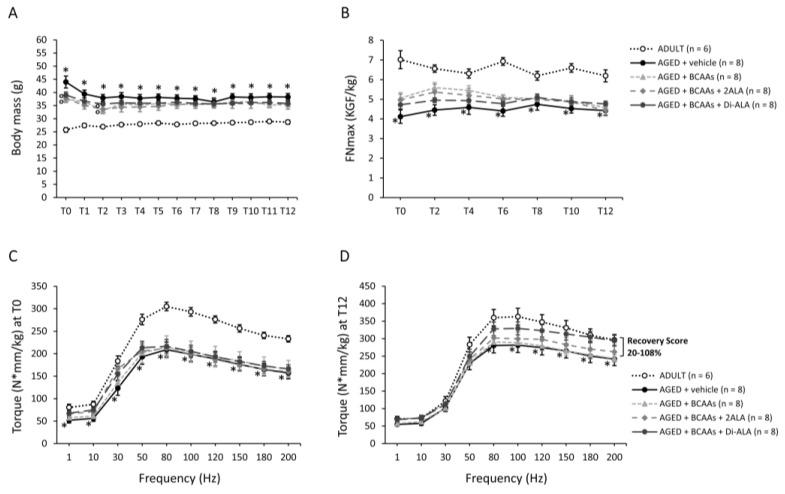
Data for body mass (BM; g), monitored once a week (from T0 to 12) in all mice cohorts (ADULT mice and AGED mice treated with vehicle, BCAAs, BCAAs + 2ALA or BCAAs + Di-ALA), and forelimb grip strength, measured once every two weeks and normalized to each animal’s BM (FNmax; KGF/kg), are shown in (**A**,**B**), respectively. All values are expressed as mean ± SEM for the number of mice indicated in brackets. For both indices, a statistically significant difference was found via an unpaired Student’s t-test for AGED mice + vehicle vs. ADULT mice at all time points (*; 0.0001 < *p* < 0.005). For BM, a statistically significant difference was found among AGED mice groups via a one-way ANOVA at T0 and T2 (F < 3.4, *p* = 0.03). Dunnett’s post hoc test, used to compare each mixture-treated group to the vehicle group, is as follows: ° vs. AGED + vehicle (0.02 < *p* < 0.04). (**C**,**D**) show values for hind limb plantar flexor torque produced at increasing frequencies (1–200 Hz), normalized to each animal’s BM (N·mm/kg), obtained from all mice groups at T0 and T12, and expressed as mean ± SEM for the number of mice indicated in brackets. A statistically significant difference was found via an unpaired Student’s t-test for AGED + vehicle vs. ADULT mice for T0 at all frequencies (*; 0.0005 < *p* < 0.01) and for T12 at frequencies from 80 to 200 Hz (0.02 < *p* < 0.05). No statistically significant differences were found among AGED mice groups via a one-way ANOVA followed by Dunnett’s post hoc test. The range of recovery scores towards ADULT values, calculated at T12 for mice treated with BCAAs + 2ALA or Di-ALA, is indicated at the right end of the lines.

AGED mice were significantly heavier than ADULT controls at all time points. For all AGED cohorts, body mass (BM; g) values resulted in homogeneously higher values at T0 and then remained constant over time, with no variations induced by any formulation (Figure 1A). Similarly, AGED mice showed lower forelimb grip strength vs. ADULT ones at each time point, whereas no significant difference was found among old mice groups, regardless of whether they were treated or not (Figure 1B).

Isometric plantar flexor torque (N·mm/kg) was measured at the start (T0, Figure 1C) and the end (T12, Figure 1D) of the treatment protocol. Both at T0 and T12, ADULT mice produced the highest torque–frequency curve, with untreated AGED mice showing significantly lower values at all frequencies (1–200 Hz) for T0 and from 80 Hz onwards for T12. The greater distance between the two curves observed at T0 is attributable to the higher BM of AGED mice at the initial time point. Notably, torque–frequency curves from all AGED cohorts, overlapping at T0 (Figure 1C), at T12 showed a trend towards increasing in mice treated with BCAAs + Di-ALA and, to a lesser extent, in those treated with BCAAs + 2ALA, with the R.S. towards ADULT values ranging between 20% and 108% at each frequency (Figure 1D).

**Figure 2:**
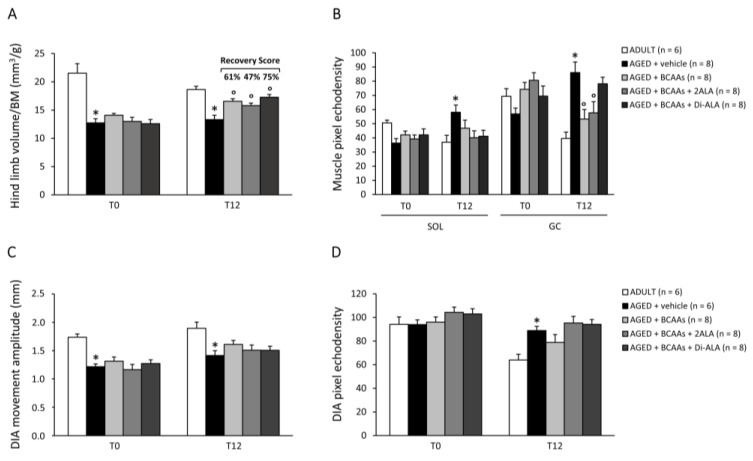
(**A**) shows BM-normalized hind limb volume (mm^3^/g), measured by ultrasonography (US) in all mice cohorts at T0 and T12. Values are expressed as mean ± SEM for the number of mice indicated in brackets. A statistically significant difference was found via an unpaired Student’s t-test for AGED + vehicle vs. ADULT mice, both at T0 and T12 (*; *p* < 0.0002). At T12, a statistically significant difference was found among AGED mice groups via a one-way ANOVA (F < 10.03, *p* < 0.0001). Dunnett’s post hoc test is as follows: ° vs. AGED + vehicle (0.0001 < *p* < 0.009). The recovery score towards ADULT value, calculated for each treated group, is indicated above the bars. Similarly, mean pixel echodensity was measured for soleus (SOL) and gastrocnemius (GC) muscles for all mice groups, as shown in (**B**). At T12, for both muscles, a statistically significant difference was found via an unpaired Student’s t-test for AGED + vehicle vs. ADULT mice (*; 0.0003 < *p* < 0.01). For GC muscle, a statistically significant difference was found among AGED mice groups via a one-way ANOVA (F = 5.06, *p* = 0.007). Dunnett’s post hoc test is as follows: ° vs. AGED + vehicle (*p* < 0.02). The values for diaphragm (DIA) movement amplitude and mean pixel echodensity, measured by US at T0 and T12 in all mice cohorts, are shown in (**C**,**D**), respectively. Values are expressed as mean ± SEM for the number of mice indicated in brackets. A statistically significant difference was found via an unpaired Student’s t-test for AGED + vehicle vs. ADULT mice, at T0 and T12 for amplitude (*; 0.0001 < *p* < 0.006), and at T12 for echodensity (*p* < 0.002). No statistically significant differences were found among AGED mice groups via a one-way ANOVA followed by Dunnett’s post hoc test.

Either at T0 or T12, untreated AGED mice showed a significant reduction in hind limb volume normalized to BM (mm^3^/g) compared to ADULT controls, measured by ultrasonography (Figure 2A). At T12, all mixtures significantly improved this index in AGED mice, with the highest R.S. (75%) observed in the BCAAs + Di-ALA group (Figure 2A). The mean pixel echodensity of SOL and GC muscles was significantly higher in AGED vs. ADULT mice at T12 (Figure 2B). A trend towards reduction in SOL muscle echodensity was observed in AGED mice treated with each formulation (Figure 2B, left); a significant reduction was found in GC muscles for mice treated with BCAAs, either alone or plus 2ALA, and a decreasing trend was observed in those treated with BCAAs + Di-ALA (Figure 2B, right). AGED mice exhibited a significant decline in DIA movement amplitude, both at T0 and T12 (mm; Figure 2C), and a significant increase in echodensity at T12 vs. ADULT controls (Figure 2D); however, no effect of any formulation was observed on these indices.

### 3.2. Ex Vivo Data

#### 3.2.1. Weight of Main Limb Muscles, Vital Organs, and Body Fat

AGED mice displayed a significant reduction in the BM-normalized (mg/g) weight of GC, tibialis anterior (TA) and quadriceps (QUAD) muscles vs. ADULT ones, whereas no appreciable differences were observed for SOL, EDL, and triceps (TRI) muscles or for vital organs. No significant changes in muscles and organ weights were observed in treated animals. BM-normalized white, but not brown, adipose tissue (WAT, BAT; mg/g) was significantly increased in AGED vs. ADULT mice, with no effect of the formulations (Table S1).

#### 3.2.2. Evaluation of Myofiber Size and Muscle Histopathology

**Figure 3:**
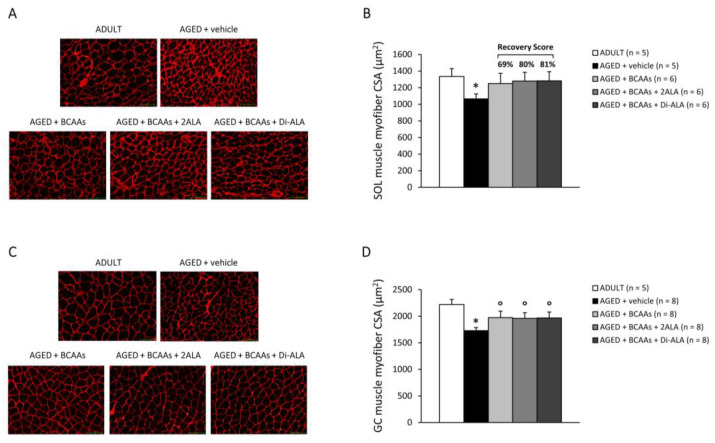
(**A**) shows representative SOL muscle sections (20× magnification) stained by immunofluorescence (IF) for laminin for each experimental group (ADULT mice and AGED mice treated with vehicle, BCAAs, BCAAs + 2ALA or BCAAs + Di-ALA). (**B**) shows the mean cross-sectional area (CSA, µm^2^) for all fiber types ± SEM, obtained from the number of mice indicated in brackets. Representative images for GC muscle (20× magnification) and mean CSA values ± SEM are shown in (**C**,**D**), respectively. For both muscles, a statistically significant difference was found via an unpaired Student’s t-test for AGED + vehicle vs. ADULT mice (*; 0.003 < *p* < 0.04). For SOL muscle, no statistically significant differences were found among AGED groups via a one-way ANOVA followed by Dunnett’s post hoc test. The recovery scores for ADULT values, calculated for each treated group, is indicated above the bars. For GC muscle, a statistically significant difference among AGED mice groups was found via a one-way ANOVA (F = 3.4, *p* < 0.03). Dunnett’s post hoc test is as follows: ° vs. AGED + vehicle (*p* < 0.03).

Immunofluorescence staining for laminin highlighted a significant reduction in the myofiber CSA (µm^2^) of SOL and GC muscles from untreated AGED vs. ADULT mice (Figure 3A–D). Treated mice displayed a comparable increment in SOL muscle myofiber CSA, with a high R.S. ranging from 69% for BCAAs to 81% for BCAAs + Di-ALA (Figure 3B). For GC muscle, a statistically significant increase in CSA was found in all treated groups vs. untreated ones (Figure 3D). Minor effects, if any, were observed in EDL muscle (Figure S1A,B). Similarly, the histological evaluation via H&E and Masson’s trichrome for SOL, GC, EDL, and DIA evidenced only a modest increase in unhealthy tissue percentage and collagen deposition in AGED mice with limited differences, if any, among the experimental groups (Table S2).

#### 3.2.3. Contractile Parameters of Isolated Muscles

**Figure 4:**
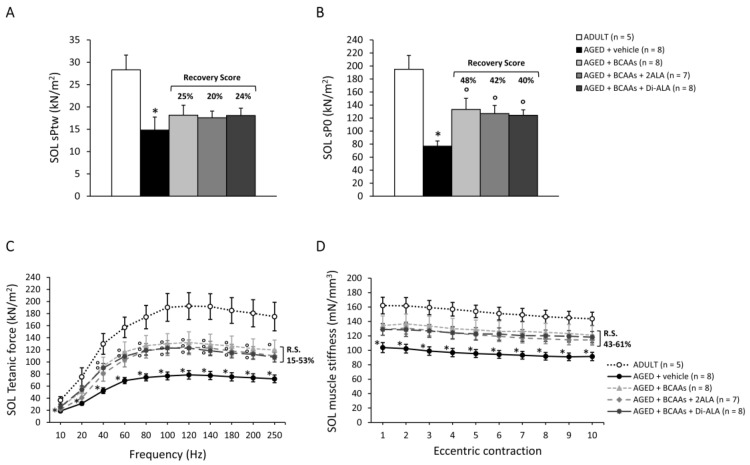
Maximal specific isometric twitch (**A**, sPtw; kN/m^2^) and tetanic (**B**, sP0; kN/m^2^) force, tetanus–frequency curve produced at increasing frequencies (10–250 Hz, **C**; kN/m^2^), and elastic properties in response to a series of 10 eccentric pulses (**D**, stiffness; mN/mm^3^) measured in SOL muscles isolated from ADULT mice and AGED mice treated with vehicle, BCAAs, BCAAs + 2ALA or BCAAs + Di-ALA. All values are expressed as mean ± SEM for the number of mice indicated in brackets. For all parameters, a statistically significant difference was found via an unpaired Student’s t-test for AGED + vehicle vs. ADULT mice (*; 0.0001 < *p* < 0.01). A statistically significant difference among AGED mice groups was found via a one-way ANOVA for sP0 (F = 4.8, *p* = 0.008) and for tetanus–frequency curve at 40 Hz and higher (F > 3.3, *p* < 0.04). Dunnett’s post hoc test, used to compare each mixture-treated group to the vehicle group, is as follows: ° vs. AGED + vehicle (0.006 < *p* < 0.05). The recovery scores (R.S.) towards ADULT values, calculated for each treated group, are indicated above the bars in (**A**,**B**) or at the right end of the lines in (**C**,**D**).

**Figure 5:**
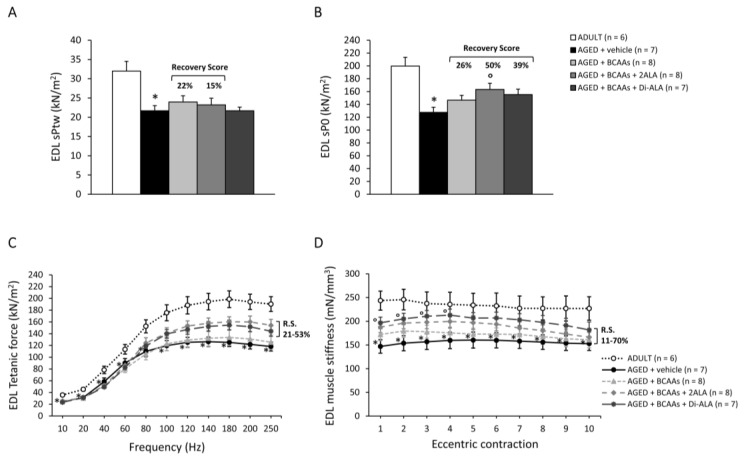
Maximal specific isometric twitch (**A**, sPtw; kN/m^2^) and tetanic (**B**, sP0; kN/m^2^) force, tetanus–frequency curve produced at increasing frequencies (10–250 Hz, **C**; kN/m^2^), and elastic properties in response to a series of 10 eccentric pulses (**D**, stiffness; mN/mm^3^) measured in EDL muscles isolated from ADULT mice and AGED mice treated with vehicle, BCAAs, BCAAs + 2ALA, or BCAAs + Di-ALA. All values are expressed as mean ± SEM for the number of mice indicated in brackets. For all parameters, a statistically significant difference was found via an unpaired Student’s t-test for AGED + vehicle vs. ADULT mice (*; 0.0003 < *p* < 0.05). A statistically significant difference among AGED mice groups was found via a one-way ANOVA for sP0 (F = 3.15, *p* = 0.04) and for stiffness from eccentric pulses from 1 to 4 (F > 2.7, *p* < 0.05). The recovery scores (R.S.) towards ADULT values, calculated for each treated group, are indicated above the bars in (**A**,**B**) or at the right end of the lines in (**C**,**D**).

**Figure 6:**
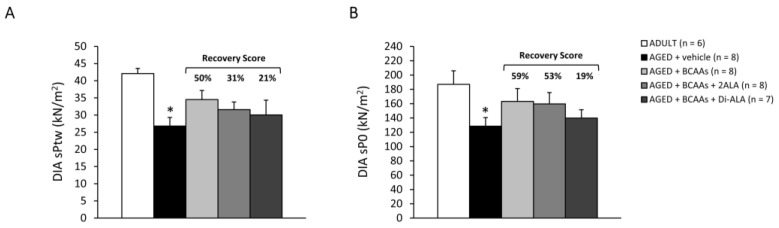
Maximal specific isometric twitch (**A**, sPtw; kN/m^2^) and tetanic (**B**, sP0; kN/m^2^) force measured in DIA muscles isolated from ADULT mice and AGED mice treated with vehicle, BCAAs, BCAAs + 2ALA or BCAAs + Di-ALA. All values are expressed as mean ± SEM for the number of mice indicated in brackets. For both parameters, a statistically significant difference was found via an unpaired Student’s t-test for AGED + vehicle vs. ADULT mice (*; 0.0004 < *p* < 0.01). No statistically significant differences were found among AGED mice groups via a one-way ANOVA followed by Dunnett’s post hoc test. The recovery scores (R.S.) towards ADULT values, calculated for each treated group, are indicated above the bars.

Data from contractile recordings performed in isolated SOL, EDL and DIA muscles from all mice cohorts are shown in Figure 4 (SOL), Figure 5 (EDL) and Figure 6 (DIA). Isometric contraction parameters of slow-twitch SOL muscle resulted in severe impairment in untreated AGED mice, as shown by the significant reduction in maximal specific twitch (Figure 4A; sPtw, in kN/m^2^) and tetanic force (Figure 4B; sP0, in kN/m^2^), as well as by the significantly lower tetanus–frequency curve (Figure 4C) compared to ADULT mice. Notably, all formulations exerted a protective effect on SOL muscle force in AGED mice, with an increasing trend in sPtw (R.S.: 20–25%), paralleled by a significant increase in sP0 and by higher tetanus–frequency curves, with a statistically significant difference vs. untreated mice from the frequency of 40Hz onwards (Figure 4A–C). Similarly, SOL muscle compliance to stretch, in response to a series of 10 eccentric stimuli (stiffness; mN/mm^3^), was significantly diminished in AGED animals and was partially rescued by each treatment, with the R.S. ranging between 43% and 61% (Figure 4D), thus indicating a positive effect of the formulations on muscle elasticity. Similarly, fast-twitch EDL muscles from untreated AGED mice showed a significant decrease in sPtw, sP0, tetanus–frequency curve and stiffness, with a partial protection (R.S. up to 70%) exerted by the mixtures, particularly those containing 2ALA or Di-ALA (Figure 5A–D). In line with ultrasonography data, a significant decrease in sPtw and sP0 vs. the ADULT group was also observed in DIA respiratory muscle; a partial amelioration was exerted by the mixtures, particularly for BCAAs and BCAAs + 2ALA (R.S. up to 59%) (Figure 6A,B).

#### 3.2.4. Biomarkers of Protein Synthesis, Immune Response, and Muscle Damage

**Figure 7:**
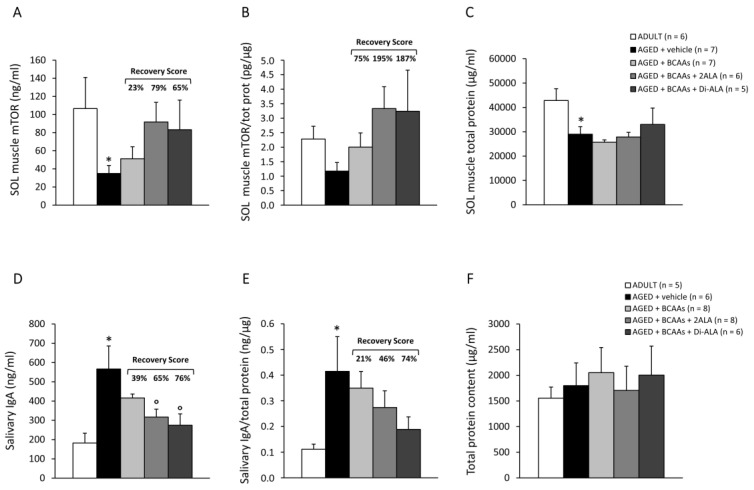
mTOR protein levels, measured by ELISA in SOL muscle from ADULT mice and AGED mice treated with vehicle, BCAAs, BCAAs + 2ALA or BCAAs + Di-ALA, expressed as absolute (**A**; ng/mL) or normalized to total protein (**B**; pg/µg). Total protein content was measured in all mice groups via a Bradford assay, and the results (µg/mL) are shown in (**C**). All values are expressed as mean ± SEM for the number of mice indicated in brackets. For A and C, a statistically significant difference was found via an unpaired Student’s t-test for AGED + vehicle vs. ADULT mice (*p* < 0.05). No statistically significant differences were found among AGED groups via a one-way ANOVA followed by Dunnett’s post hoc test for any parameter. The recovery scores towards ADULT values, calculated for each treated group, are indicated above the bars. (**D**,**E**) show immunoglobulin A (IgA) levels measured in saliva samples collected from all mice, expressed as absolute (ng/mL) or normalized to salivary total protein (ng/µg). Total protein content was measured via a Bradford assay, and the results (µg/mL) are shown in (**F**). Values are expressed as mean ± SEM for the number of mice indicated in brackets. For (**D**,**E**), a statistically significant difference was found via an unpaired Student’s t-test for AGED + vehicle vs. ADULT mice (*; 0.02 < *p* < 0.05). Only for absolute IgA values, a statistically significant difference among AGED mice groups was found via a one-way ANOVA (F = 3.87, *p* < 0.03). Dunnett’s post hoc test is as follows: ° vs. AGED + vehicle (*p* < 0.02). Recovery scores are indicated above the bars.

mTOR protein levels (ng/mL, Figure 7A), measured via ELISA in SOL muscle, were significantly lower in untreated AGED mice vs. ADULT controls. This difference was maintained, although in a non-statistically significant manner, after normalization to the total protein content (Figure 7B). This latter index (Figure 7C) was significantly reduced in AGED mice vs. ADULT ones, with no modifications induced by the treatments. However, the formulations, particularly the 2ALA and Di-ALA-enriched ones, increased both absolute and normalized mTOR levels in AGED mice, with an R.S. of up to 195%.

Untreated AGED mice displayed significantly increased levels of salivary IgA, which were either absolute (ng/mL, Figure 7D) or normalized to the total protein content (ng/µg, Figure 7E; total protein in µg/mL, Figure 7F), in line with previous observations concerning an imbalanced humoral immunity in elderly patients [29]. The formulations, particularly the ones containing 2ALA or Di-ALA, effectively reduced the release of IgA in saliva, with R.S. ranging from 46% to 76%; the decrease was indeed statistically significant for absolute levels (Figure 7D,E).

CK and LDH plasma levels (U/L), indicators of muscle damage and metabolic sufferance, respectively, did not evidence substantial modifications in mice groups (Table S3).

## 4. Discussion

With estimates that, by mid-century, one in six people globally will be aged ≥ 65 years (https://www.un.org/development/desa/pd/, accessed on 01 December 2022), the prevalence of sarcopenia will inevitably rise. Although no “silver bullet” exists to fight this condition, the current body of research indicates that lifestyle interventions, among which there are nutritional ones, are core strategies for the management of sarcopenia [11,12]. The alterations in skeletal muscle protein turnover and balance observed in older subjects [5,14] highlight the need of an adequate protein intake [13], with supplements favoring muscle anabolism and limiting catabolism, especially for high-quality oral nutritional supplements containing EAAs.

In this setting, our study aimed to provide further preclinical evidence to the still-limited data on the benefits of BCAAs—considered the best amino acids in terms of anabolic properties—in sarcopenia [15,16,17,18,19], and to support that novel oral formulations containing 2ALA or Di-ALA can boost some BCAAs effects observed in muscle-wasting conditions [20], also in the context of aging. Overall, our data strongly support the validity of the naturally AGED murine model for preclinical studies on sarcopenia, particularly in the chosen age window (17–20 months).

In vivo, all AGED mice cohorts exhibited a stably and significantly higher body mass in comparison to ADULT controls, directly related to the observed increase in visceral and subcutaneous WAT, also not modified by any mixture. This agrees with the age-related shift in visceral fat extensively reported in old mice [30] and humans [31].

Importantly, our ultrasound evaluation showed that AGED mice, who were either 17- or 20-months-old, had a significantly reduced hind limb volume—a clear atrophy indicator—with the supplements efficiently counteracting this decrease. In parallel, the muscle-specific examination of main hind limb plantar flexors, i.e., GC and SOL, highlighted a significant increase in ultrasound echodensity in untreated AGED mice compared to ADULT controls, being partially lowered by the formulations. The modest increment, if any, in collagen content detected by Masson’s trichrome in either GC or SOL muscles from AGED mice suggests the contribution of different components, i.e., fat and fibrous tissue, in age-related increases in echodensity [28,32].

Moreover, we confirmed the severe functional impairment of the whole plantar flexor muscle group, since a significant decrease in torque–frequency curves was observed in AGED mice already at T0 (i.e., 17-month-old animals); this can, in part, be related to impaired neuromuscular drive and motor unit changes, identified as players in sarcopenia [33]. In 20-month-old mice, the formulations, especially the one combining BCAAs and Di-ALA, induced a pronounced recovery of this in vivo index, and minor effects were found on the impairment of in vivo forelimb grip strength, measured in non-anesthetized animals. In vivo functional assessments were influenced by multiple factors other than muscle force, e.g., the concerted function of vascular and nervous systems, and by animal behavior, which may have different susceptibility to BCAAs supplementation [23].

In line with these considerations, ex vivo muscle physiology confirmed a significant age-associated decline in isometric force and compliance to stretching after eccentric stimulation in both SOL and EDL muscles, in line with previous reports [34,35]. These indices were significantly improved by all formulations in AGED SOL muscle and were partially ameliorated in EDL muscle.

At the morphological level, although the mixtures did not protect hind limb muscles from weight decline, both SOL and GC muscles displayed an increase in mean myofiber CSA—significantly reduced by aging—in response to treatments. By contrast, these differences could not be appreciated in EDL muscle. Paired with functional data, this further suggests a more pronounced sarcopenic phenotype at the level of lower limb weight-bearing muscles in AGED mice, as previously observed in HU mice [20]. In addition, and in line with previous studies [20,21], BCAAs are expected to have a greater effect in muscles composed mainly of slow-twitch mitochondrial oxidative myofibers, also in relation to the activity of branched-chain amino acid transaminase 2, the mitochondrial enzyme involved in the muscle-specific metabolism of BCAAs [36].

In this view, SOL muscle was chosen to assess the impact of each formulation on protein synthesis by measuring both total protein and mTOR protein levels. AGED mice displayed a significant reduction in total protein, paralleled by significantly decreased mTOR levels. This is consistent with the decline in mTOR signaling pathway efficiency and its impact on the loss of proteostasis in aging muscles [2]. The formulations, particularly the ones containing 2ALA or Di-ALA, partially restored mTOR protein expression in SOL muscle, although this was not sufficient to overcome total protein decline. This discrepancy could be possibly explained by the condition of anabolic resistance described in old C57BL/6J mice and in human aging, which may, at least in part, have hindered the ability of the formulations to restore adequate protein levels [5,14]. In this regard, combination with other interventions, such as physical exercise and protein-enriched foods, may help to overcome anabolic resistance in this setting.

Functional and structural alterations in the DIA—defined as respiratory sarcopenia—have been described in naturally aged mice and associated with increased susceptibility to respiratory complications in the elderly [37,38]. In vivo, our ultrasound evaluation disclosed that AGED mice had a significantly reduced DIA movement amplitude, which was consistent with alterations in respiratory function recently observed by whole-body plethysmography [39]. The functional impairment of AGED DIA was confirmed by a significant decrease in isometric force, without parallel remarkable histopathological alterations in or signs of fibrosis [37,39]. Only a partial functional benefit was observed with BCAAs-based formulations in AGED DIA muscle, supporting the hypothesis of a preferential action on specific muscle groups, which requires further investigation.

Finally, our data show that salivary IgA levels are increased in AGED mice, corroborating the imbalanced immune response observed in elderly people, which may be secondary to a deficient activity of regulatory T cells and consequent hyperfunction of B lymphocytes [29]. Interestingly, formulations containing 2ALA or Di-ALA significantly reduced the release of IgA in saliva, confirming their ability to differently modulate this accessible biomarker of humoral immunity in various physiopathological contexts characterized by low-grade chronic inflammation [20,21].

## 5. Conclusions

This work corroborates the validity of naturally aged mice for preclinical studies on sarcopenia with translational value to the human aging process, in a context where the identification of treatments to overcome sarcopenia is of utmost importance from a global health perspective. Overall, our results support the usefulness of oral supplementation with BCAAs for possibly improving muscle health in sarcopenic conditions, and they confirm the ability of novel formulations containing L-Alanine, especially as a dipeptide (Di-ALA), to boost BCAAs action on specific disease-relevant readouts, particularly in SOL and GC muscles, as previously demonstrated in other muscle-wasting conditions [20]. These findings pave the way to an adequate use of these nutritional supplements in age-related sarcopenia, more likely not just as a single intervention, but in the frame of patient-tailored treatments considering the multifactorial complexity of sarcopenia.

In this context, the greater variability of human nutrition vs. controlled animal studies needs to be considered. In particular, any indication for BCAAs-based supplement integration should be defined after a careful evaluation of protein intake and balance, also in relation to nutrition style or high-protein diet regimens, in order to obtain the expected benefit and the best cost-effectiveness ratio.

## 6. Patents

Amino acid mixtures were provided by Dompé farmaceutici S.p.A. with patent applications no. 102019000010401 (BCAAs + 2ALA) and WO2020260689 (BCAAs + Di-ALA).

## Figures and Tables

**Table 1:** Composition and daily final dose (mg/kg) for each tested formulation.

Formulation	Composition: BCAAs + ALA (Weight Ratio of L-Leu:L-Ile:L-Val:L-ALA/Di-ALA)	Final Dose (mg/kg)
BCAAs	2:1:1	656
BCAAs + 2ALA	2:1:1:2	984
BCAAs + Di-ALA	2:1:1:2	984

## Data Availability

The data presented in this study are available on request from the corresponding author.

## Supplementary Materials

The following supporting information can be downloaded at https://www.mdpi.com/article/10.3390/nu15020330/s1: Figure S1: Evaluation of myofiber size in EDL muscle. Table S1: Weight of main limb muscles, vital organs, and body fat. Table S2: Muscle histopathology. Table S3: Plasma levels of biochemical markers of muscle damage.
