# Supplementary material for: Branched-Chain Amino Acids and Di-Alanine Supplementation in Aged Mice: A Translational Study on Sarcopenia
Source: Nutrients. 2023 Jan 9;15(2):330. doi: 10.3390/nu15020330 (PMC9861351; doi:10.3390/nu15020330)
Supplement: Supplementary file 1 [file nutrients-15-00330-s001.zip › nutrients-2121711-supplementary.pdf]

Supplementary materials

Branched-chain amino acids and Di-Alanine supplementation in aged mice: a translational study on sarcopenia

Paola Mantuano[1], Brigida Boccanegra[1], Gianluca Bianchini[2], Ornella Cappellari[1], Lisamaura Tulimiero[1], Elena Conte[1], Santa Cirmi[1], Francesca Sanarica[1], Michela De Bellis[1], Antonietta Mele[1], Antonella Liantonio[1], Marcello Allegretti[2], Andrea Aramini[2*], Annamaria De Luca[1*]

[1] Section of Pharmacology, Department of Pharmacy-Drug Sciences, University of Bari "Aldo Moro", Orabona 4 - Campus, 70125 Bari, Italy

[2] Research & Early Development, Dompé farmaceutici S.p.A., Via Campo di Pile, s.n.c. 67100 L'Aquila, Italy

* Correspondence to: annamaria.deluca@uniba.it; tel.: +39 080 5442245; andrea.aramini@dompe.com; tel.: +39 0862 338340

Figure S1

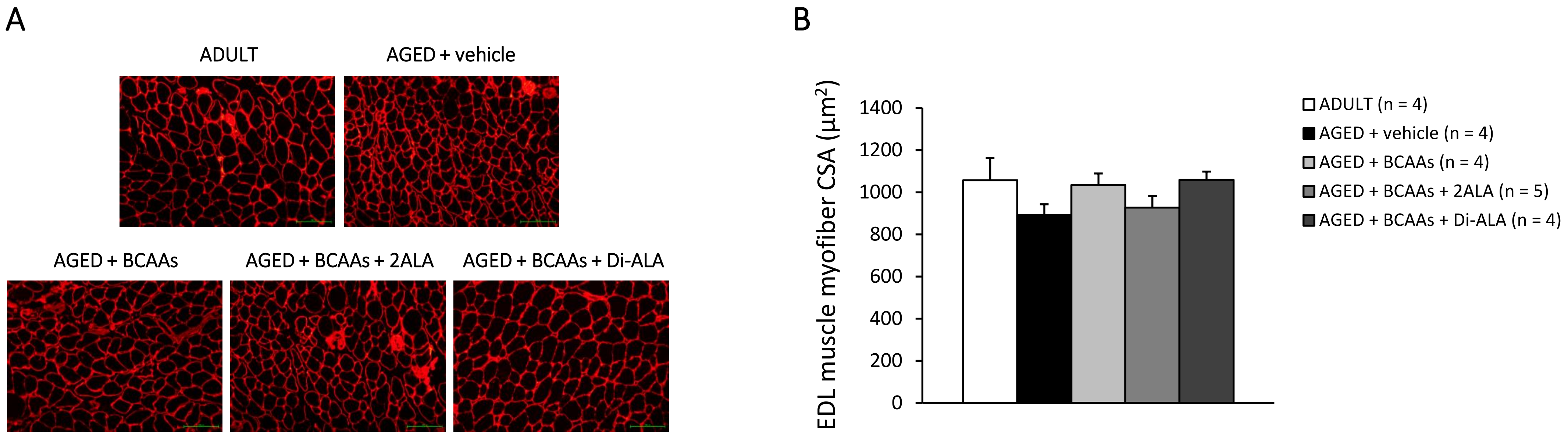

Figure S1. Evaluation of myofiber size in EDL muscle

(**A**) shows representative EDL muscle sections (20× magnification) stained by immunofluorescence (IF) for laminin, for each experimental group (ADULT mice and AGED mice treated with vehicle, BCAAs, BCAAs + 2ALA, or BCAAs + Di-ALA). (**B**) shows the mean cross-sectional area (CSA, μm^2) for all fiber types ± SEM, obtained from the number of mice indicated in brackets. No statistically significant differences were found for AGED + vehicle vs. ADULT mice via an unpaired Student's t-test, or among AGED groups via a one-way ANOVA followed by Dunnett's post hoc test.

Table S1

Group	Weight of hind limb muscles/BM (mg/g)						Weight of vital organs/BM (mg/g)					Body fat (mg/g)	
	TA	GC	QUAD	SOL	EDL	TRI	Liver	Heart	Kidneys	Spleen	Brain	WAT	BAT
ADULT (n = 6)	1.73 ± 0.04	5.07 ± 0.1	7.42 ± 0.17	0.28 ± 0.01	0.35 ± 0.02	3.66 ± 0.16	53.4 ± 3.2	5.64 ± 0.33	6.95 ± 0.19	3.11 ± 0.25	15.5 ± 0.39	12.0 ± 1.2	3.05 ± 0.40
AGED + vehicle (n = 8)	1.40 ± 0.06 *	4.14 ± 0.17 *	5.92 ± 0.24 *	0.25 ± 0.01	0.36 ± 0.03	3.63 ± 0.13	56.4 ± 2.4	5.12 ± 0.16	7.84 ± 0.28	2.49 ± 0.11	11.8 ± 0,27	19.7 ± 2.2 *	3.76 ± 0.53
AGED + BCAAs (n = 8)	1.45 ± 0.06	4.53 ± 0.18	6.58 ± 0.23	0.28 ± 0.02	0.33 ± 0.01	3.64 ± 0.26	50.4 ± 1.8	5.17 ± 0.35	7.37 ± 0.33	2.82 ± 0.34	11.7 ± 1.5	17.3 ± 1.8	3.73 ± 0.69
AGED + 2ALA (n = 8)	1.45 ± 0.04	4.51 ± 0.16	6.16 ± 0.29	0.26 ± 0.03	0.33 ± 0.01	3.63 ± 0.18	53.0 ± 2.0	5.45 ± 0.28	7.07 ± 0.28	2.34 ± 0.10	13.3 ± 0.51	18.6 ± 3.5	3.58 ± 0.38
AGED + Di-ALA (n = 8)	1.49 ± 0.05	4.51 ± 0.09	5.99 ± 0.16	0.26 ± 0.02	0.30 ± 0.02	3.43 ± 0.17	55.6 ± 2.0	5.20 ± 0.15	7.25 ± 0.15	2.26 ± 0.11	12.3 ± 0.4	17.5 ± 2.4	3.41 ± 0.40

Table S1. Weight of main limb muscles, vital organs, and body fat

Weight of tibialis anterior (TA), gastrocnemius (GC), quadriceps (QUAD), soleus (SOL), extensor digitorum longus (EDL), and triceps (TRI) muscles, vital organs (liver, heart, kidneys, spleen, and brain, and white and brown adipose tissue (WAT, BAT), normalized to mice body mass (BM; mg/g), harvested from ADULT mice and AGED mice treated with vehicle, BCAAs, BCAAs + 2ALA, or BCAAs + Di-ALA. All values are expressed as mean ± SEM from the number of mice indicated in brackets. For TA, GC, and QUAD muscles, a statistically significant difference was found via an unpaired Student's t-test for AGED + vehicle vs. ADULT mice (*; $p < 0.001$). For WAT, a statistically significant difference was found via an unpaired Student's t-test for AGED + vehicle vs. ADULT mice (*; $p < 0.02$). No statistically significant differences were found among AGED mice groups via a one-way ANOVA followed by Dunnett's post hoc test.

Table S2

Group	H&E staining Unhealthy tissue (%)				Masson's trichrome staining Collagen (%)			
	SOL	GC	EDL	DIA	SOL	GC	EDL	DIA
ADULT (n = 4 – 6)	11.1 ± 1.8	2.78 ± 0.64	9.15 ± 1.7	16.3 ± 1.6	2.43 ± 0.22	4.07 ± 0.56	2.87 ± 0.49	9.13 ± 0.70
AGED + vehicle (n = 4 – 8)	14.6 ± 0.95	3.21 ± 0.48	11.9 ± 2.8	12.8 ± 0.91	4.97 ± 2.3	5.17 ± 1.0	5.20 ± 1.3	8.36 ± 1.2
AGED + BCAAs (n = 4 – 8)	14.6 ± 1.9	4.16 ± 0.86	12.9 ± 1.9	14.2 ± 1.4	4.70 ± 1.1	4.21 ± 0.71	6.03 ± 1.6	7.74 ± 0.84
AGED + 2ALA (n = 4 – 8)	13.3 ± 2.3	4.62 ± 0.55	13.9 ± 1.7	15.8 ± 1.4	5.38 ± 2.3	4.53 ± 0.86	7.16 ± 1.0	8.89 ± 0.48
AGED + Di-ALA (n = 4 – 8)	14.5 ± 1.3	4.53 ± 0.59	12.2 ± 1.8	14.5 ± 0.94	5.65 ± 2.4	4.31 ± 0.68	5.89 ± 1.2	10.8 ± 1.5

Table S2. Muscle histopathology

On the left, the percentage (%) of unhealthy tissue (*i.e.*, fibrotic/necrotic/regenerated areas) on total muscle area, quantified via classical haematoxylin and eosin (H&E) staining; on the right, the percentage (%) of collagen on total muscle area, quantified via Masson's trichrome staining, in SOL, GC, EDL, and DIA muscles sections from ADULT mice and AGED mice treated with vehicle, BCAAs, BCAAs + 2ALA, or BCAAs + Di-ALA. All values are expressed as mean ± SEM from the number of mice indicated in brackets. No statistically significant differences were found for AGED + vehicle vs. ADULT mice via an unpaired Student's t-test, or among AGED groups via a one-way ANOVA followed by Dunnett's post hoc test.

Table S3

Group	Plasma biomarkers	
	CK (U/L)	LDH (U/L)
ADULT (n = 6)	2176 ± 434	902 ± 120
AGED + vehicle (n = 8)	5047 ± 2041	1361 ± 265
AGED + BCAAs (n = 8)	4715 ± 1955	1377 ± 378
AGED + 2ALA (n = 8)	4615 ± 1044	1733 ± 564
AGED + Di-ALA (n = 8)	5651 ± 1728	1356 ± 274

Table S3. Plasma levels of biochemical markers of muscle damage

Levels of creatine kinase (CK) and lactate dehydrogenase (LDH) measured by spectrophotometry in plasma samples from ADULT mice and AGED mice treated with vehicle, BCAAs, BCAAs + 2ALA, or BCAAs + Di-ALA. All values are expressed as mean ± SEM from the number of mice indicated in brackets. No statistically significant differences were found for AGED + vehicle vs. ADULT mice via an unpaired Student's t-test, or among AGED groups via a one-way ANOVA followed by Dunnett's post hoc test.
